# Intermittent Fasting Reduces Intestinal Inflammation in Dextran Sulfate Sodium‐Induced Colitis of Mice

**DOI:** 10.1002/fsn3.70014

**Published:** 2025-02-02

**Authors:** Shuo Song, Xiwen Zhang, Haoyue Zheng, Yun Liao, Ping Tang, Yu Liu, Aifa Tang, Pixin Ran, Xizhuo Sun, Pingchang Yang

**Affiliations:** ^1^ Department of General Practice Medicine Third Affiliated Hospital of Shenzhen University Shenzhen China; ^2^ Institute of Allergy & Immunology Shenzhen University School of Medicine and State Key Laboratory of Respiratory Diseases Allergy Division at Shenzhen University Shenzhen China; ^3^ Shenzhen Clinical School of Medicine Guangzhou University of Chinese Medicine Shenzhen China; ^4^ State Key Laboratory of Respiratory Diseases, National Clinical Research Center for Respiratory Disease, Guangzhou Institute of Respiratory Health Guangzhou Medical University Guangzhou China

**Keywords:** inflammation, inflammatory bowel disease, intermittent fasting, intestine

## Abstract

Inflammatory bowel disease (IBD), comprising ulcerative colitis (UC) and Crohn's disease (CD), is a chronic condition impacting both the gastrointestinal tract and the immune system. Intestinal inflammation and epithelial injury are the pathological features of IBD. Recent studies have reported that some strategies of dietary restriction (DR) can regulate immune system, correct the immune disorders, and improve some immune‐associated diseases such as IBD. However, as a form of DR, the effect of intermittent fasting (IF) on the IBD remains unknown. In this study, we investigated the therapeutic efficacy of two cycles of IF on the IBD mouse model induced by dextran sulfate sodium (DSS). It was found that two cycles of IF significantly decreased the score of the disease activity index (DAI) and alleviated the IBD‐related symptoms. In addition, IF reversed the shortening of colon length mediated by DSS, significantly increased the number of colonic crypts, and decreased the colonic histological score. Furthermore, the proportion of CD4^+^ T cells in both the spleen and mesenteric lymph node was reduced by IF treatment. The expression of serum pro‐inflammatory cytokines IL‐1β, TNF‐α, and IL‐6 was restrained by IF intervention. Moreover, IF administration significantly reduced the number of leukocytes and macrophages infiltrating around the crypt base in the colon. In conclusion, these results demonstrated that IF administration can alleviate the symptoms and pathology of IBD in the DSS‐induced IBD mouse model by reducing the intestinal inflammation.

## Graphical abstract

The DSS‐induced colitis mouse model was established to evaluate the ameliorative properties of IF intervention. The study found that two cycles of IF significant alleviate the symptoms and pathology of IBD in colitis mouse model by reducing intestinal inflammation. The therapeutic effects of IF on IBD were achieved solely by intermittent calorie restriction and were not associated with the nutrient component, offering a novel approach for the treatment of IBD.

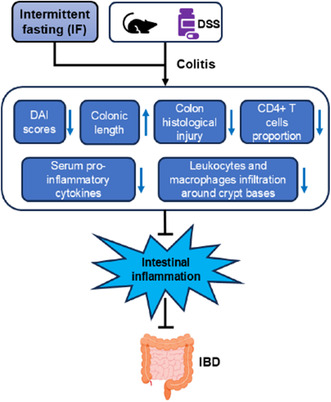

## 1 Introduction

The prevalence of Inflammatory Bowel Disease (IBD) has been steadily increasing in recent years, presenting a significantly global healthcare challenge (Xavier and Podolsky 2007). Ulcerative a colitis (UC) and Crohn's disease (CD) are two forms of IBD distinguished by acute and chronic inflammation in the intestine. IBD is an autoimmune disease that can also result in complications that are life‐threatening (Zhang et al. 2020). Although the etiology of IBD remains unclear, more and more evidence suggests that genetics, intestinal microbiota, environmental factors, and the immune system all play vital roles in the pathogenesis of IBD (Podolsky 2002; Danese and Fiocchi 2006). Dietary factors are an important factor of environmental factors, and they are involved in the global spread of IBD, such as the popularity of Western diets increasing the risk of IBD worldwide (Kaplan and Ng 2017).

Recent studies reveal that the categories and levels of nutrients can affect the generation, survival, and function of lymphocytes and have an impact on several autoimmune diseases (Choi, Lee, and Longo 2017). Dietary restriction (DR) has the potential to prevent or reverse immune disorder through eliminating autoimmune cells and activating hematopoietic stem cells (HSC)‐dependent regeneration while minimizing the side effects (Tang et al. 2016; Mihaylova, Sabatini, and Yilmaz 2014; Cheng et al. 2014). The major DR regimens comprise intermittent fasting (IF), caloric restriction (CR), time‐restricted feeding (TRF), and fasting‐mimicking diet (FMD) (Fontana, Partridge, and Longo 2010; Fontana and Partridge 2015; Chaix et al. 2014; Brandhorst et al. 2015). IF is a form of DR that alternates eating and fasting periods. Previous studies have shown that IF can lead to body weight loss, improving insulin sensitivity, lowering blood pressure, attenuating inflammation, and counteracting oxidative stress (Bhutani et al. 2013; Carter, Clifton, and Keogh 2016; Eshghinia and Mohammadzadeh 2013; Johnson et al. 2007).

We have demonstrated that FMD has an obvious improvement on IBD in mice by reducing intestinal inflammation and promoting the regeneration and repair of intestinal epithelium (Song et al. 2021). Longo's group has also reported that FMD can improve IBD‐associated symptoms through reducing intestinal inflammation, promoting intestinal stem cells (ISCs) renewal, and increasing beneficial gut microbiota (Rangan et al. 2019). Our FMD intervention regimen was limited to the calorie intake per mouse that was 30% of the control group for three consecutive days and followed by a 4‐day normal chow *ad libitum*. The FMD was characterized by low levels of carbohydrates and proteins but high levels of dietary fiber. It is necessary to investigate whether improving IBD is a result of the nutrient component or calorie restriction of FMD, or both factors involved. In order to address this problem, we designed an IF intervention regimen to investigate the effect on the IBD mouse model. During IF intervention period, the calorie intake of each mouse was restricted to 30% of the control group, but the nutrient component of the IF diet was as the same as the normal chow.

In this study, we found that two cycles of 2‐day IF treatment could reduce the histological damage in the colon, decrease CD4^+^ T cells proportion in the spleen and mesenteric lymph nodes, and lower the level of serum pro‐inflammatory cytokines IL‐1β, TNF‐α, and IL‐6, reducing the infiltration of leukocytes and macrophages around crypt bases, resulting in alleviating the symptoms and pathology of IBD in mice. The study indicates for the first time that IF, which is only associated with intermittent calorie restriction and not related to the nutrient component, can significantly alleviate the symptoms and pathology associated with IBD in the mouse model and offers a novel approach for the treatment of IBD.

## 2 Methods and Materials

### 2.1 Mouse Models and Dietary Regimens

The animal experimental protocols received approval from the Institutional Animal Care and Use Committee (IACUC) at Shenzhen University with an approval number of IACUC‐202300103. 6‐week‐old female C57BL/6 mice were obtained from the Guangdong Experimental Animal Center (Guangdong, China). The mice were housed in a specific pathogen‐free (SPF) barrier facility with consistent temperature and humidity at the Laboratory Animal Center of Shenzhen University. The mice were kept in an environment where the light and dark cycle was controlled for 12 h each. All mice were randomly assigned to three groups: control group, DSS group, and DSS + IF group. Mice in the DSS group and DSS + IF group were given 2.5% w/w DSS (Yeasen Biotechnology, Shanghai, China) as their exclusive drinking water for 5 days and, followed by 7‐days of purified water in three cycles. The IF was administrated starting on the 8th day and ended on the 9th day in cycle 2 and cycle 3. Each mouse in DSS + IF was individually housed to ensure that the calorie intake was restricted to 30% of that in the control group during the IF intervention. The standard rodent chow *ad libitum* was provided to all of the mice except the mice in DSS + IF group during IF intervention.

### 2.2 Disease Activity Index (DAI) Scores

The Disease Activity Index (DAI) was utilized for the evaluation of IBD severity, encompassing assessments for body weight reduction, stool consistency, and presence of rectal bleeding. The DAI scores of each mouse were recorded daily during cycle 2 and cycle 3. The DAI scores for body weight loss were categorized as follows: 0, absence of body weight decrease; 1, a decrease of 1%–5% in body weight; a decrease of 5%–10% in body weight; 3, a decrease of 10%–20% in body weight; 4, an excess of 20% reduction in body weight. The DAI scores for stool consistency were classified as follows: 0, solid pellets; 1, adherent in pellet form and soft; 2, stool loose and with some firmness; 3, liquid consistency evident and stool loose; 4, acute diarrhea. The rectal bleeding was assessed by direct observation and recording of visible blood in the rectum and stools, while the existence of an occult blood in stools was determined using occult blood test strip (W.H.P.M. Bioresearch & Technology, Beijing, China). The scoring of rectal bleeding in the DAI was as follows: 0, absence of hemoccult; 1, presence of hemoccult; 2, visual pellet bleeding along with positive hemoccult; 3, positive hemoccult accompanied by the presence of observable blood in stool and rectal bleeding; 4, the hemoccult test is positive with a visually significant fecal occult blood and concurrent rectal bleeding. The DAI scores were calculated based on stool consistency and hemoccult but not the body weight loss because there was a rapid reduction in body weight following calorie restriction during IF intervention, and the loss in body weight did not impact the health of mice.

### 2.3 Colon H&E Staining Histological Scoring

After the mice were euthanized by cervical dislocation, we harvested the proximal part of the colon. Colon tissues underwent fixation in 4% paraformaldehyde for a duration of 24 h at room temperature, followed by dehydration and paraffin embedding procedures. The colon tissue embedded in the sample was sectioned into 4 μm thick slices. The tissue sections were subjected to Hematoxylin & Eosin staining according to the manufacturer's instructions (Servicebio, Wuhan, China) and were captured using an Olympus microscope (Japan) at magnifications of 10× and 20×. The histological scoring of colonic mucosa is a reliable approach for evaluating the extent of inflammation and tissue injury. The criterion of histological scoring is as follows: score 0 indicates the absence of inflammation and injury to the mucosa of the colon; score 1 denotes mild edema and inflammation in the colon mucosa with removal of one‐third of the crypts; score 2 represents moderate inflammation in the mucosa with removal of two‐thirds of the crypts; score 3 signifies moderate inflammation in the mucosa with complete removal of crypts; finally, score 4 reflects severe mucosal injury in the colon.

### 2.4 Flow Cytometry

We obtained the immune cells in the spleen and mesenteric lymph nodes by grinding and filtering through a 70 μm filter and separated the immune cells from suspension through centrifugation. The lysis buffer (BD Bioscience) was used to lyse the erythrocytes in the spleen. We used the antibodies APC‐Cy7 CD3e (BD Biosciences, USA), PE CD4, and PE‐Cy CD8a in the experiment. In the first step, cells were treated with dye of BV510 for 30 min to exclude the nonviable cells and then washed with PBS to eliminate the residual dye. The second step was to stain the immune cells using the antibodies to CD3e, CD4, and CD8 for 30 min. For the last stage, the cells were subjected to a washing procedure and were subsequently resuspended in PBS. The strategy of gating was as follows: Firstly, BV510 negative cells were gated to pick out the live cells. Secondly, T cells were gated by which were CD3‐positive cells. Finally, helper T cells and cytotoxic T cells were gated by which were CD4^+^ and CD8^+^ T cells, respectively. The sample measurement was performed using the CytoFLEX LX cytometer (Beckman Coulter), and the data was processed with FlowJo software for analysis.

### 2.5 Enzyme‐Linked Immunosorbent Assay (ELISA)

We used serum separation tubes to collect mice blood; the blood samples were coagulated at room temperature for 30 min and then centrifuged for 15 min at 1000 × g to get serum samples. The serum was stored at −80°C until the time to detect. The serum levels of IL‐1β, TNF‐α, IL‐6, and IFN‐γ were measured by ELISA commercial reagent kits (Enzyme‐linked Biotechnology, Shanghai, China). The 100 μL standard samples with different concentrations and test samples were added to reaction wells to react at room temperature for 2 h. After that, the 100 μL of biotinylated antibody working solution was added to the reaction wells, mixed gently, and covered with sealing tape, and reacted for 1 h at room temperature. Next, 100 μL of 1 × SA‐HRP working solution was added to reaction wells, covered with sealing tape and reacted for 0.5 h at room temperature. We added 50 μL color‐developing solution A to each well and then added 50 μL color‐developing solution B, covered it with sealing tape and reacted it for 15 min at room temperature. After the color reaction was finished, we added 50 μL stop solution to each well and mix gently. All of the reaction procedures were completed in a dark place. The absorbance was measured at 450 nm with a microplate reader.

### 2.6 Immunofluorescence

After deparaffinization in xylene, the colon paraffin sections were rehydrated using a gradient of ethanol solution (100%, 90%, 70%, 50%, and 30%) for 5 min at each concentration, followed by being rinsed with purified water. The antigens of colon tissues were retrieved in 0.1 M citrated buffer (pH = 6.0) at 100°C for 30 min. The antigen was subjected to a 1.5‐h incubation at room temperature in the presence of blocking buffer (3% goat serum + PBS + 0.1% Triton 100). After blocking, the sections were incubated with primary antibodies at 4°C overnight in a humidified box. The primary antibodies we used were anti‐F4/80 (1:200, Cell Signaling Technology) and anti‐CD45 (1:200, Cell Signaling Technology). The secondary antibodies were incubated in a dark box for 1 h after 24 h. The Alexa Fluor 546 IgG (1:500, Life Technologies) was used as a secondary antibody. Then, we used the Hoechst 33342 (Molecular Probes, USA) to stain the nuclei after being washed with PBS. Finally, we covered the sections with anti‐fading aqueous mounting medium (Sigma‐Aldrich, USA). The images were captured at a magnification of 20× by the Zeiss LSM 510 confocal microscope.

### 2.7 Statistical Analysis

The statistical variances between two groups were assessed through the application of Student's *t*‐test, and one‐way ANOVA was applied to analyze the statistical variances among three groups. The statistical analysis was performed by using the software of GraphPad Prism. Data were reported as mean ± SEM. The significance was established for *p* values < 0.05.

## 3 Results

### 3.1 The Symptoms Associated With IBD Can Be Alleviated With Two Cycles of IF in the Mouse Model of IBD

**FIGURE 1:**
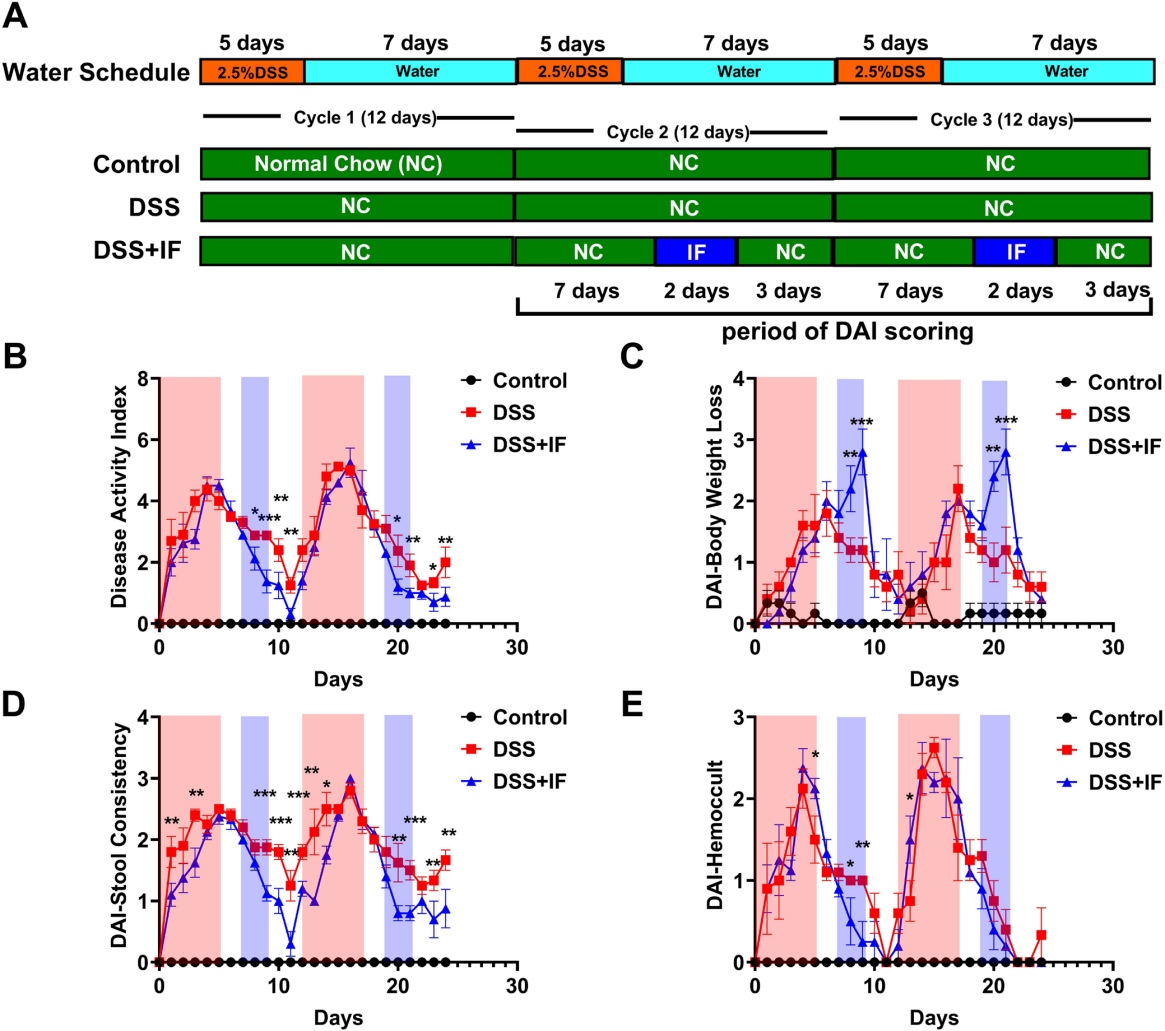
IF improves the symptoms associated with IBD and reduces DAI in the mouse model of IBD. (A) The protocol of the experiment for managing drinking and feeding in three groups. (B) The DAI score for three groups that comprise the score of stool consistency and hemoccult test during cycles 2 and 3: control group (*n* = 6), DSS group (*n* = 5), and DSS + IF group (*n* = 5). (C) DAI scores for assessing weight reduction. (D) DAI scores for assessing stool consistency. (E) DAI scores for assessing the bleeding in stool. The period of DSS treatment is represented by the red shading in (B–E), while the blue shading indicates the period of IF application. The data are displayed as the mean ± SEM; The * indicates a significant distinction between the DSS and DSS + IF groups. *p < 0.05; **p < 0.01; ***p < 0.001.

To evaluate the impact of IF on IBD, we established the IBD mouse model by DSS. Mice received 2.5% DSS in their drinking water for 5 consecutive days and, followed by 7‐days of water in each cycle. The cycle was repeated three times in total (Figure 1A). 9‐week‐old female C57BL/6 mice were randomly allocated into three groups: control group, DSS group, and DSS + IF group. The mice in the control group were fed with normal chow *ad libitum* without DSS treatment. The mice in the DSS group received three cycles of DSS treatment and were fed with normal chow *ad libitum*. The mice in the DSS + IF group received three cycles of DSS treatment and applied the IF intervention during cycles 2 and 3 (Figure 1A). In the DSS + IF group, 2‐day IF intervention was beginning on the 8th day of both cycle 2 and 3, followed by 3‐day normal chow *ad libitum* (Figure 1A). The calorie consumption of each mouse in the DSS + IF group was restricted to 30% of the control group.

The DAI of three groups was assessed daily during cycles 2 and 3. DAI includes the loss in body weight, alterations in stool consistency, and presence of blood in the stool. We modified the DAI scoring that only comprise the scores of hemoccult and stool consistency due to the rapid weight loss caused by IF. The DAI scores of the DSS + IF group exhibited obvious reduction compared to those of DSS group starting on the 8th day of cycle 2, and this difference persisted until the end of cycle 2 (Figure 1B). The significant differences was reappeared through the application of IF intervention in cycle 3 (Figure 1B). The DAI scores of the DSS + IF group for body weight loss showed a rapid decrease after IF intervention as the reduction in calorie intake (Figure 1C). The DAI scores for stool consistency exhibited a notable decrease in the DSS + IF group in comparison to the DSS group. The most obvious improvement for IF to IBD‐related symptoms was the amelioration of stool consistency (Figure 1D). The DAI scores for hemoccult in the DSS + IF group exhibited an obvious reduction subsequent to IF treatment during cycle 2 when compared to the DSS group (Figure 1E). The data manifest that IBD‐related symptoms can be alleviated by two cycles of IF in the IBD mouse model.

### 3.2 IF Maintains the Colon Length and Enhances Crypt Count but Decreases the Histological Score of the Colon

**FIGURE 2:**
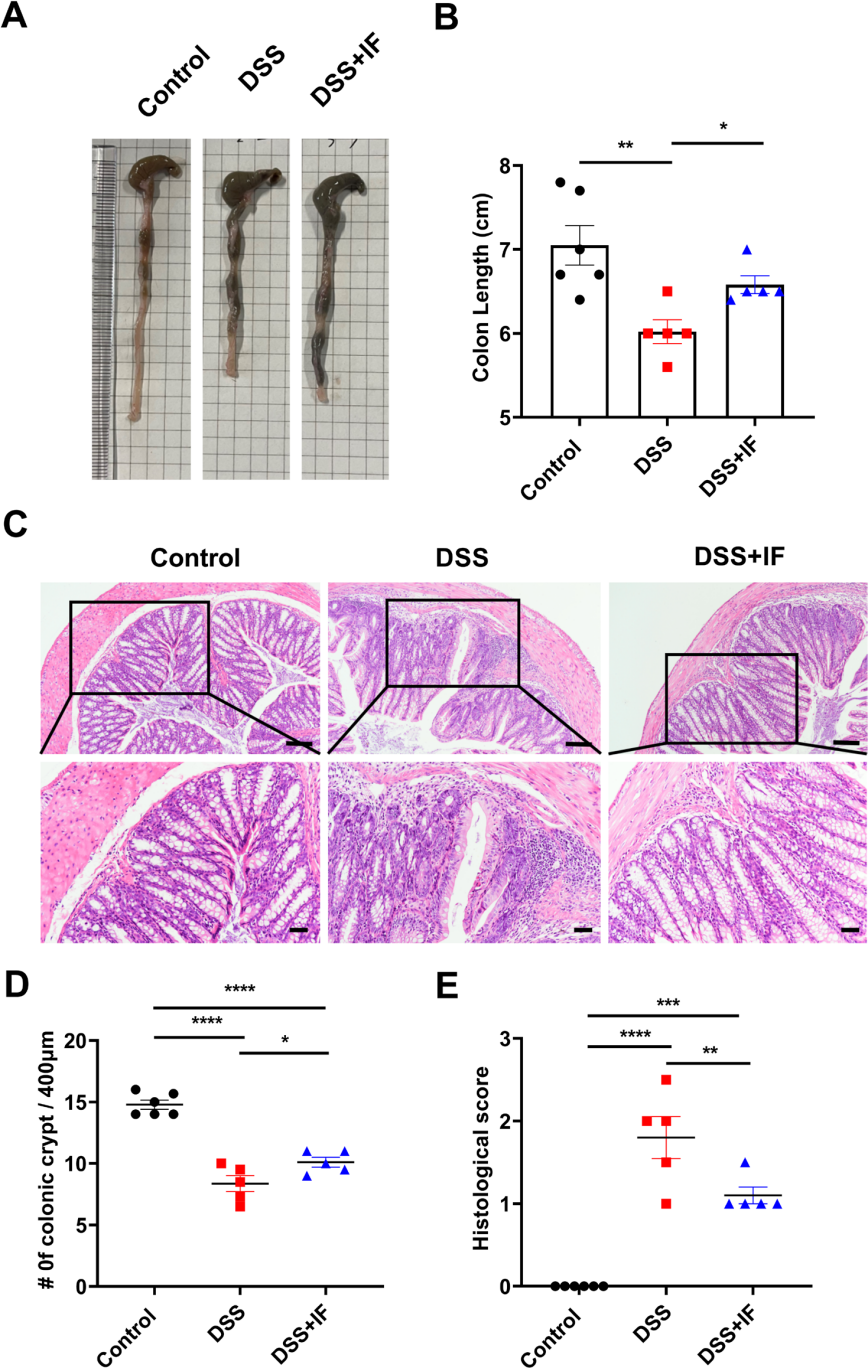
IF maintains colon length and reduces colonic inflammatory injury. (A) The representative colon images of the three groups. (B) The lengths of the colon were measured in the control group (*n* = 6), DSS group (*n* = 5), and DSS + IF group (*n* = 5). (C) The colon tissue was stained with HE, and images were captured at a magnification of 20×. The scale bars for the upper panels are 50 μm, and for the lower panels, they are 20 μm. (D) The colonic crypts number varied among the control group (*n* = 6), DSS group (*n* = 5), and DSS + IF group (*n* = 5). (E) The histological scores were evaluated on colon in the control group (*n* = 6), DSS group (*n* = 5), and DSS + IF group (*n* = 5). The data are reported as mean ± SEM; *p < 0.05; **p < 0.01; ***p < 0.001; ****p < 0.0001.

In order to evaluate the severity of inflammation in the colon, we measured the colonic length of mice in three groups (Figure 2A). The average colon length of the control group was 7.05 cm, while the average colon length of the DSS group was shortened to 6.02 cm as the DSS treatment. It is interesting to note that the average colon length of the DSS + IF group was 6.58 cm, which was significantly longer than that of the DSS group and it indicated that the shortening of colon length caused by the DSS can be reversed by IF (Figure 2B).

The integrity of the colonic epithelium was assessed by counting the number of colonic crypts (Figure 2C). The DSS + IF group showed a notable increase in the number of crypts when compared to the DSS group (Figure 2D). In order to assess the degree of injury in the colon, we used colon HE staining sections for histological scoring. DSS + IF group exhibited a notably lower score in comparison to the DSS group, which showed that there was lesser damage in the colon of the DSS + IF group compared to the DSS group (Figure 2C,E). The results indicate that IF can significantly reduce the damage caused by DSS in the colon.

### 3.3 IF Reduces T Cells Proportion in the Spleen and Mesenteric Lymph Nodes

**FIGURE 3:**
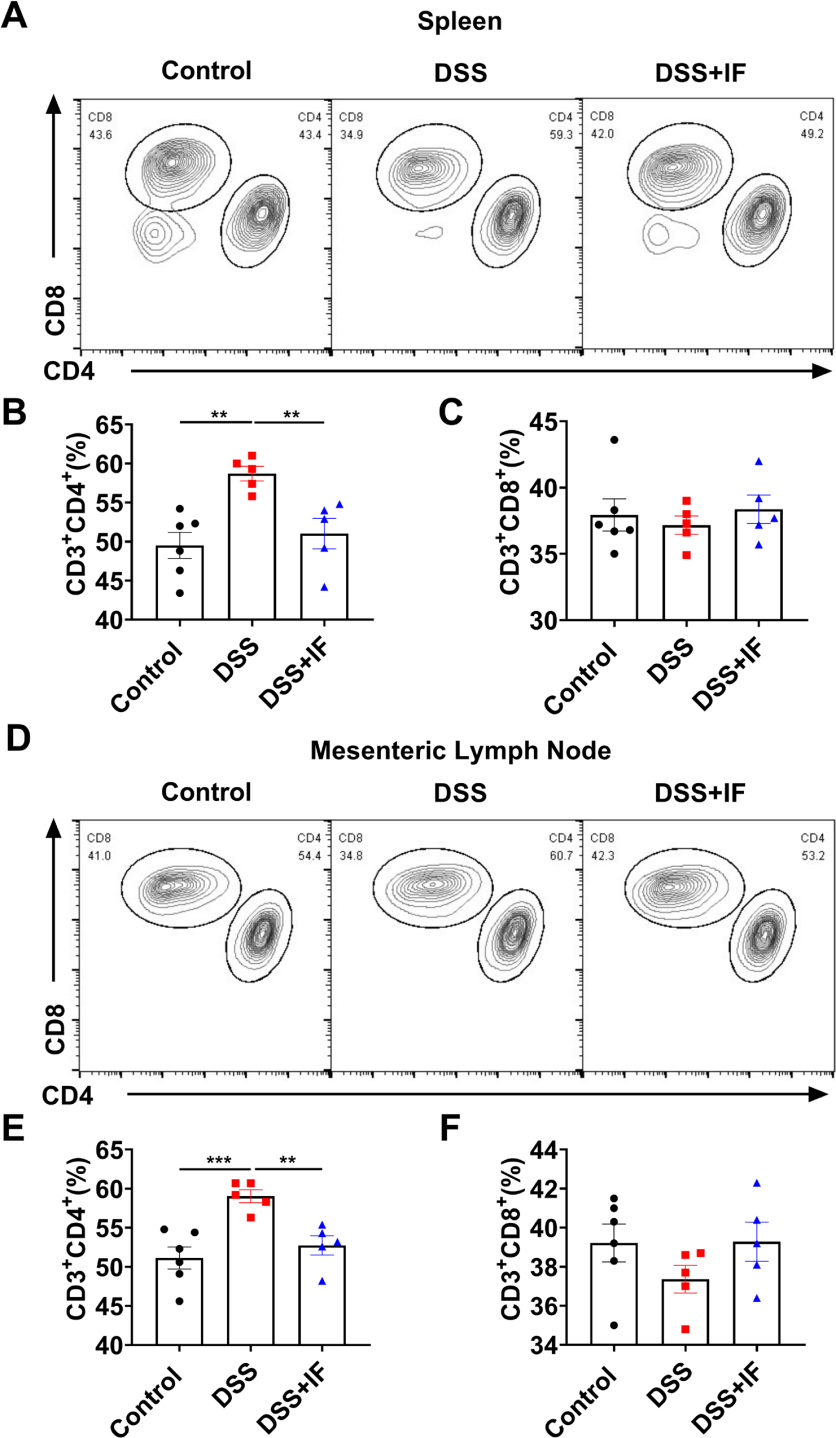
IF reduces T cells proportion in the spleen and mesenteric lymph node. (A) The outcomes of spleen T cells proportion in the control group (*n* = 6), DSS group (*n* = 5), and DSS + IF group (*n* = 5) were detected by flow cytometry. (B) The comparison of spleen CD4^+^ T cell proportion among the control group, DSS group, and DSS + IF group. (C) The comparison of spleen CD8^+^ T cell proportion among the control group, DSS group, and DSS + IF group. (D) The outcomes of mesenteric lymph node T cell proportion in the control group (*n* = 6), DSS group (*n* = 5), and DSS + IF group (*n* = 5). (E) The comparison of mesenteric lymph node CD4^+^ T cells proportion among the control group, DSS group, and DSS + IF group. (F) The comparison of mesenteric lymph node CD8^+^ T cell proportions among the control group, DSS group, and DSS + IF group. The data are reported as mean ± SEM; **p < 0.01; ***p < 0.001.

We used flow cytometry to detect the proportional change of CD4^+^ and CD8^+^ T cells in the spleen (Figure 3A). We noted a marked reduction in the proportion of CD4^+^ T cells in the spleen of the DSS + IF group as compared to the DSS group (Figure 3B). There was no obvious difference in the proportion of CD8^+^ T cells among the three groups (Figure 3C). We also analyzed the proportional change of CD4^+^ and CD8^+^ T in mesenteric lymph nodes (Figure 3D). In comparison to the DSS group, the DSS + IF group exhibited a notable reduction in CD4^+^ T cells (Figure 3E). The proportion of CD8^+^ T cells in mesenteric lymph nodes did not show obvious variances among the three groups (Figure 3F). The results suggest that IF reduces the proportion of CD4^+^ T cells in both the spleen and mesenteric lymph nodes.

### 3.4 IF Restrains the Expression of Pro‐Inflammatory Cytokines in the Serum

**FIGURE 4:**
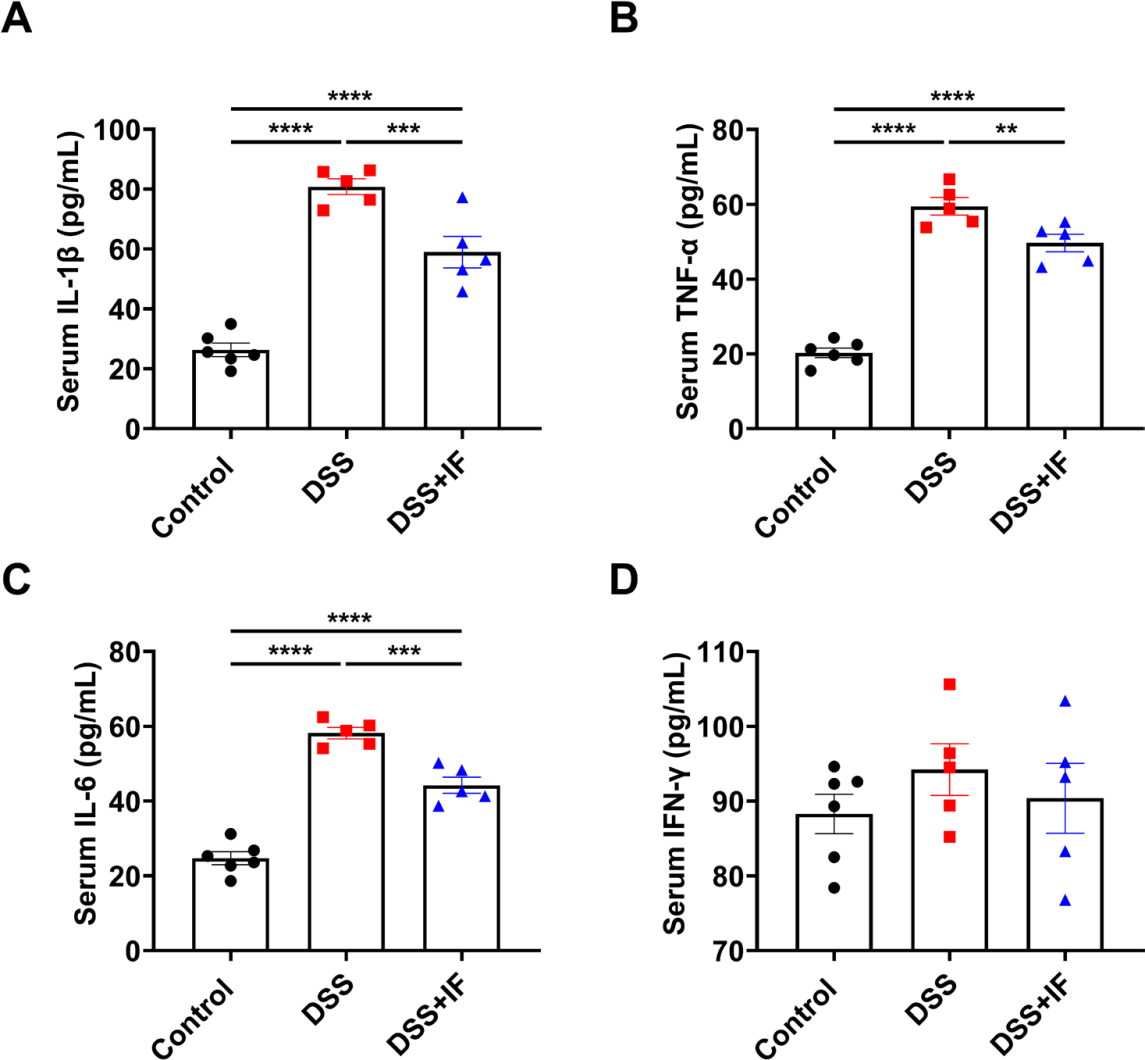
IF restrains the expression of pro‐inflammatory cytokines in the serum. (A) The level of serum IL‐1β (pg/mL) in the control group (*n* = 6), DSS group (*n* = 5), and DSS + IF group (*n* = 5). (B) The level of serum TNF‐α (pg/mL) in the control group (*n* = 6), DSS group (*n* = 5), and DSS + IF group (*n* = 5). (C) The level of serum IL‐6 (pg/mL) in the control group (*n* = 6), DSS group (*n* = 5), and DSS + IF group (*n* = 5). (D) The level of serum IFN‐γ (pg/mL) in the control group (*n* = 6), DSS group (*n* = 5), and DSS + IF group (*n* = 5). The data are reported as mean ± SEM; **p < 0.01; ***p < 0.001; ****p < 0.0001.

Cytokines play a vital role in the pathogenesis of IBD, and the imbalance between pro‐inflammatory and anti‐inflammatory cytokines in IBD leads to the intestinal inflammation and tissue damage (Neurath 2014). The elevated levels of pro‐inflammatory cytokines are related to the severity of IBD (Neurath 2014). We found a significant decrease in the serum cytokine level of interleukin‐1β (IL‐1β) in the DSS + IF group compared with the DSS group and observed a similar reduction in the level of serum tumor necrosis factor alpha (TNF‐α) in DSS + IF group when compared to DSS group (Figure 4A,B). Interleukin‐6 (IL‐6) is a keystone cytokine that has pro‐inflammatory properties in the progression of IBD (Hunter and Jones 2015) and we observed that the serum IL‐6 level in the DSS + IF group was significantly decreased when compared to the DSS group (Figure 4C). Interferon gamma (IFN‐γ) is a pleiotropic cytokine that has a crucial role in mediating a series of immune responses (Ng, Fong, and Abdullah 2023). Interestingly, there was no obvious difference in the level of serum IFN‐γ among the three groups (Figure 4D). These results indicate that IF reduced the level of serum pro‐inflammatory cytokines in IBD mouse model.

### 3.5 IF Decreases Leukocytes and Macrophages Infiltration Around Colonic Crypt Bases

**FIGURE 5:**
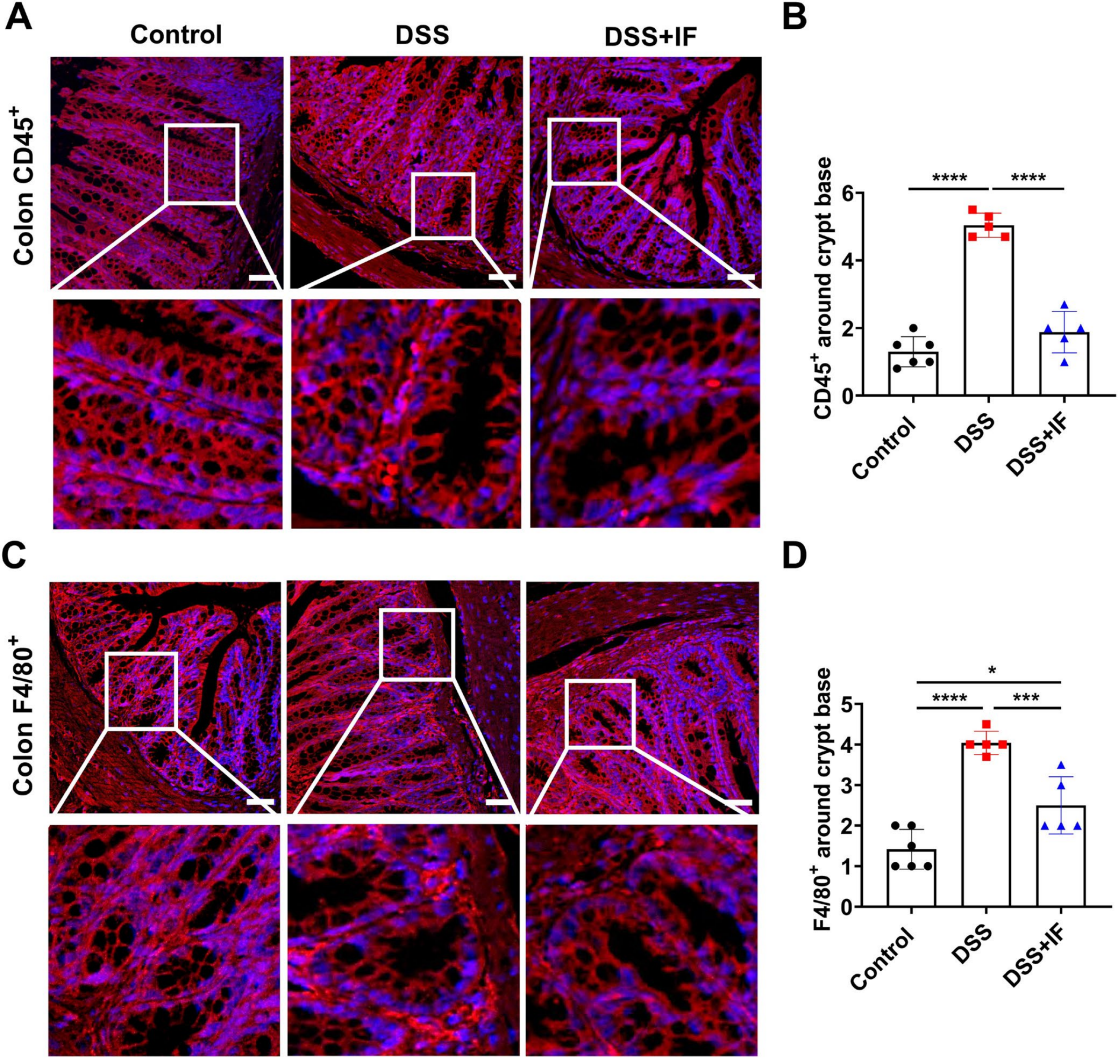
IF reduces immune cell infiltration around colonic crypt bases. (A) The colonic immunofluorescence staining representative images for CD45^+^ cells in the control group (*n* = 6), DSS group (*n* = 5), and DSS + IF group (*n* = 5). (B) The quantitation of CD45^+^ cells around colonic crypt bases. (C) The colonic immunofluorescence staining representative images for F4/80^+^ cells in the control group (*n* = 6), DSS group (*n* = 5), and DSS + IF group (*n* = 5). (D) The quantitation of F4/80^+^ cells around colonic crypt bases. The data are reported as mean ± SEM; *p < 0.05; ***p < 0.001; ****p < 0.0001. The images for immunofluorescence staining were captured at 20× magnification. The scale bar measures 50 μm.

To evaluate the severity of inflammation in the colon of mice, we utilized immunofluorescence analysis to detect the inflammatory markers in the colon. Through immunofluorescence analysis, we observed a notable reduction in the quantity of CD45^+^ cells (a marker of leukocytes) around the crypt base in the DSS + IF group as compared to the DSS group (Figure 5A,B). In addition, the number of F4/80^+^ cells (a marker of macrophages) surrounding the crypt base in the DSS + IF group was also reduced as compared to the DSS group (Figure 5C,D). These data show that IF can reduce macrophage and leukocyte infiltration that is surrounding the crypt base in the colon of the IBD mouse model and offer the evidence that IF has a strong effect on attenuating inflammation in the colon.

## 4 Discussion

The generation and functions of immune cells can be influenced by the type and component of nutrients, as indicated by recent studies (Choi, Lee, and Longo 2017). Nutrients and metabolism are the primary regulators of the immune system, and the types of dietary restrictions can affect several autoimmune diseases (MacIver, Michalek, and Rathmell 2013). In our studies, we found that two cycles of IF reduced DAI scores and relieved the IBD‐related symptoms in the mouse model of IBD. Through histological analysis, we revealed that the number of crypts was raised and the damage degree of intestinal mucosa was decreased by IF intervention. The shortening of the colon was reversed by IF application under the DSS treatment, which indicates that IF may repress inflammatory response and damage of the colon. The pathogenesis of IBD is significantly influenced by the involvement of T cells (Xavier and Podolsky 2007). The proportion of CD4^+^ T cells in both the spleen and mesenteric lymph node was decreased by IF according to the result of flow cytometry. The data shows that IF can decrease the systemic inflammation in the IBD mouse model. Moreover, the expression of serum pro‐inflammatory cytokines IL‐1β, TNF‐α, and IL‐6 was restrained by IF intervention. Additionally, we observed an obvious decrease in the number of macrophages and leukocytes surrounding the colonic crypt base following two cycles of IF administration, which indicated that IF can decrease the activation and recruitment of immune cells in the lamina propria (LP) of the colon. All in all, these results show that two cycles of IF can decrease both intestinal and systemic inflammation in the IBD mouse model.

Both we and Longo's group have reported that FMD has a significant alleviating effect on IBD in mice, but the nutrient components of FMD that we and Longo's group used are different from the normal chow (Song et al. 2021; Rangan et al. 2019). The component of FMD we used during intervention is characterized by low levels of carbohydrates and protein but high level in dietary fiber, and the component of Longo's FMD used is low level of carbohydrates but high in fat and dietary fiber (Song et al. 2021; Rangan et al. 2019). In this study, the component of IF we used was the same as normal chow, and the alleviating effect is only associated with the intermittent calorie restriction. Our result indicates for the first time that IF, which is only associated with intermittent calorie restriction, can significantly alleviate the symptoms and pathology associated with IBD in the mouse model. Another different point is that the total time of IF intervention was shortened to 2 days followed by 3‐days of normal chow, while the total time of our FMD was a 3‐day FMD intervention followed by 4‐days of normal chow, and the total time of Longo's FMD was a 4‐day FMD intervention followed by 2‐days of normal chow. The shortening of total time for our IF regimen is beneficial for overcoming the poor compliance of intermittent calorie restriction and promoting the clinical transformation of IF for IBD treatment. The study also is the first time to show that the level of IL‐1β, TNF‐α, and IL‐6 in the serum of colitis mice was greatly decreased by IF. The IL‐1β, TNF‐α, and IL‐6 are pro‐inflammatory cytokines that can promote inflammation and enhanced pathogen elimination, and the lower level of pro‐inflammatory represents the lesser inflammatory response and fewer inflammatory injuries.

This study has revealed that IF has an obvious effect on the therapy of IBD and attenuates the intestinal inflammation by intermittent calorie restriction. There are some recent studies reporting that the immune cell dynamic and immune responses could be impacted by the conversion of fasting and refeeding. For example, calorie restriction can promote memory T cell accumulation in the bone marrow (BM), and the memory T cells homing to BM during calorie restriction is related to the protection against infections and tumors (Collins et al. 2019). In another study, it was discovered that the ketone bodies (KBs), which were induced by fasting, can regulate CD8^+^ T cell metabolism, enhance CD8^+^ T cells bioenergetics and cytokine production, resulting in optimizing the CD8^+^ T cell effector response to bacterial infection and cancer (Luda et al. 2023). In addition, a study found that fasting can induce circulating monocytes to migrate to the BM, and the re‐feeding results in a surge of monocytes into circulation, and it alters the immune response to bacterial infection (Janssen et al. 2023). All in all, these studies indicate that fasting and calorie restriction have a strong impact on immune cells and the immune system, and the underlying mechanisms are complex and deserve to be explored in the future.

There are some limitations of the study. Our data suggest that the application of two‐cycle IF intervention can alleviate the symptoms and pathology of IBD, in mice by reducing systemic and intestinal inflammation. The dietary factor is an important factor for the pathogenesis of IBD and we present a new method to improve IBD‐associated symptoms in mice by intermittent calorie restriction, but another factor, such as intestinal flora and the changes and effect of intestinal flora after IF treatment, is unclear, so it is necessary to detect the condition of mice intestinal flora in the later work. Another limitation is lacking the data that is associated with humans. Although we found that the intestinal inflammation and pathology can be significantly decreased in the IBD mouse model, there are many differences between humans and mice, and these data only support that the IF has potential therapeutic effects on the IBD for humans so it needs more data from randomized clinical trials to support the therapeutic effect of IF on IBD. In addition, we did not investigate the mechanism of how IF reduces the systemic and intestinal inflammation in the IBD mouse model, and the underlying molecular mechanism is important to be explored in the future.

In conclusion, these findings suggest that IF can relieve the symptoms and pathology of IBD in a mouse model by reducing systemic and intestinal inflammation. The therapeutic effects of IF on IBD were achieved solely by intermittent calorie restriction and were not associated with the nutrient component, offering a novel approach for the treatment of IBD.

## Author Contributions

**Shuo Song:** data curation (lead), formal analysis (lead), investigation (lead), methodology (lead), project administration (lead), software (lead), writing – original draft (lead). **Xiwen Zhang:** investigation (supporting), methodology (supporting), validation (supporting). **Haoyue Zheng:** investigation (supporting), methodology (supporting), validation (supporting). **Yun Liao:** investigation (supporting), methodology (supporting). **Ping Tang:** investigation (supporting), methodology (supporting). **Yu Liu:** methodology (supporting), resources (supporting). **Aifa Tang:** investigation (supporting), methodology (supporting), resources (supporting). **Pixin Ran:** conceptualization (equal). **Xizhuo Sun:** conceptualization (equal). **Pingchang Yang:** conceptualization (lead), funding acquisition (lead), methodology (supporting), resources (lead), supervision (lead), validation (equal), writing – review and editing (lead).

## Ethics Statement

The experimental protocols involved were all approved by the Institutional Animal Care and Use Committee (IACUC) of Shenzhen University with an approval number of IACUC‐202300103.

## Conflicts of Interest

The authors declare no conflicts of interest.

## Data Availability

All data are included in this article. Any additional questions should be directed contacted to the corresponding author.
